# A Frequency Domain Analysis of the Excitability and Bifurcations of the FitzHugh–Nagumo Neuron Model

**DOI:** 10.1021/acs.jpclett.1c03406

**Published:** 2021-11-05

**Authors:** Juan Bisquert

**Affiliations:** Institute of Advanced Materials (INAM), Universitat Jaume I, 12006 Castelló, Spain

## Abstract

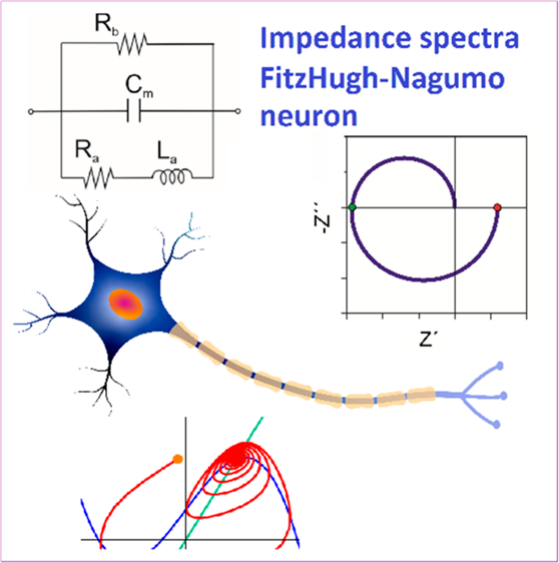

The dynamics of neurons consist of oscillating patterns of a membrane potential that underpin the operation of biological intelligence. The FitzHugh–Nagumo (FHN) model for neuron excitability generates rich dynamical regimes with a simpler mathematical structure than the Hodgkin–Huxley model. Because neurons can be understood in terms of electrical and electrochemical methods, here we apply the analysis of the impedance response to obtain the characteristic spectra and their evolution as a function of applied voltage. We convert the two nonlinear differential equations of FHN into an equivalent circuit model, classify the different impedance spectra, and calculate the corresponding trajectories in the phase plane of the variables. In analogy to the field of electrochemical oscillators, impedance spectroscopy detects the Hopf bifurcations and the spiking regimes. We show that a neuron element needs three essential internal components: capacitor, inductor, and negative differential resistance. The method supports the fabrication of memristor-based artificial neural networks.

A brain is a complex structure where computing and memory are tightly intertwined, conducted by large networks of neurons connected by synapses. Individual neurons function by gated ion channels that undergo positive and negative membrane voltage feedback cycles in response to multiple incoming stimuli. The neuronal activation triggers voltage spikes of equal magnitude at a variable rate.^1^ Understanding the firing patterns of neurons and the coupled oscillations of neuronal ensembles allows us to better understand the operation of the brain. Furthermore, the neuronal firing patters are the key component of neuromorphic engineering, which aims to design and build artificial neural systems, like computational arrays of synapse-connected artificial neurons, retinomorphic vision systems, or auditory processors, using (micro)electrical components and circuits.^2,3^ Massively parallel brain-inspired in-memory computing operations may bring much needed advances in the spatial density of computational resources and power consumption and overcome the Von-Neumann bottleneck.^4−7^ These model biological systems are adaptive, fault tolerant, and scalable and process information using energy-efficient, asynchronous, event-driven methods, well suited to cognition and motor tasks. It is already possible to create functional elements for the construction of biomimetic computational systems such as spiking neural networks (SNNs) based on memristor elements,^8,9^ such as NbO spiking neuristors.^10,11^

The technique of small-amplitude impedance spectroscopy (IS) is widely used in electrochemistry and materials science for the analysis of physicochemical processes and the characterization of experimental parameters.^12^ The modulated current in response to a small periodic perturbation of voltage with angular frequency ω is measured. The resulting linear impedance data are described in terms of an equivalent circuit (EC) model that provides detailed information about the physical processes occurring at different time and frequency scales. Investigating the frequency domain response allows one to decompose the response of a device into a set of characteristic spectra that can be readily recognized. The method is used widely in biophysics^13−16^ and can be applied to memristors and neuron models.^17^

The impedance measurements were important historically for deriving the paradigm of membrane excitability by Hodgkin and Huxley (HH)^18^ that underpins the current understanding of neuronal activity.^19^ Early in the 20th century, the membrane was found to have a frequency-dependent impedance.^20,21^ The main early neuron models, such as Lapicque’s 1907 integrate-and-fire model,^22^ the 1952 HH model,^18^ and that of Nagumo et al.,^23^ analyzed the neuronal activity using the methods of electrical circuits. However, in later years the time domain methods prevailed, and the IS properties of neurons have not been systematically described, to the best of our knowledge. Here we aim to provide tools for the characterization of the dynamical properties of neurons, by analysis of a representative dynamical model in the frequency domain. We want to obtain a classification of spectra corresponding to different dynamical behaviors of the neuron.

To describe neuronal response and behavior, we adopt dynamical models formed by a nonlinear set of differential equations that emulate the actual output of a biological neuron.^24−26^ HH is a complete description of an excitable membrane by the concerted actuation of several ion channels, but it is computationally complex as it involves the membrane voltage and three different internal state variables. Simpler models can generate many dynamical properties of the neurons. A minimal dynamical model is composed of a two-dimensional system that contains the evolution of membrane potential *u* and a slower “recovery” variable *w*.^27^ The first minimal model was developed in 1961 by FitzHugh^28^ by reducing the three slow variables of the HH model to just one refractory current. Nagumo et al.^25^ formulated the model in an electrical analogy, including inductor and negative resistance elements. The resulting FitzHugh–Nagumo (FHN) model displays realistic neural dynamics like the cessation of repetitive spiking as the amplitude of the stimulus current increases.^23,27^ It has also been broadly studied by its rich phase portraits, as described in books and review articles,^27,29−31^ and it is computationally efficient for analyzing the dynamics of neural networks.^32,33^ The dynamics of systems of coupled FHN neurons and their bifurcation properties have been amply studied in recent years.^34−39^

There are several motivations for studying the impedance spectroscopy of neurons.

(1) The impedance of neurons can be measured experimentally, for the characterization of neural diseases.^40,41^ Despite the enormous advances in theory and experiments, IS has not been used in the analysis of the operation of neuronal systems.

(2) A neuron undergoes a bifurcation when it is displaced between qualitatively different states by noise or external current: from rest to a spiking oscillation consisting of repetitive firing patterns. The presence of bifurcations is a key aspect to the theory of dynamical systems in neuroscience. The characterization of bifurcations in terms of impedance response has been amply studied in oscillating electrochemical systems.^42−45^ According to standard methods of bifurcation theory,^46^ the linearized equations for a small displacement provide important information about the nature of the trajectories in a large perturbation. Because the IS model is obtained by a linear response, the bifurcation properties of a system can be signaled by the impedance spectra on the implementation of Nyquist’s stability criterion. This method has been fully developed in the field of electrochemical oscillators by Koper and others.^47−50^ A chemically oscillating system or a spiking neuron is tracing a limit cycle around an unstable fixed point.^46,51^ Therefore, both types of systems are controlled by similar fundamental processes that can be unified in EC representations. Chua and co-workers^52,53^ applied the stability criteria of IS to show that the spiking of HH model neurons occurs in unstable regions.

(3) Once the methods described above have been outlined, the IS analysis of dynamical models of neurons can become an effective guide for the design and fabrication of artificial neurons using a variety of platforms of organic and inorganic materials. Investigating the impedance response of material devices can provide information about their dynamical response. Recently,^17^ we showed the properties of IS of neuron models like HH and the adaptative integrate-and-fire model and how they relate to the halide perovskite memristor.^54^

In this paper, we describe the stability, bifurcations, and oscillations in the FHN neuron model adapted to a small signal IS method. We use the methods developed previously in the frequency domain^17^ to obtain the ac impedance of a neuron model. Here we relate for the first time the impedance spectra to the spiking time dynamics of a firing neuron, by solving simultaneously the FHN neuron in the frequency and time domain. The analysis in the frequency domain provides a new angle for the study of neurons and network dynamics. We apply Koper’s method of IS criteria for bifurcations of electrochemical oscillators^47−50^ to dynamical neuron models. Our results show the essential structure of neuromorphic oscillatory elements such as memristors for coupled spiking networks,^55,56^ in terms of the impedance properties that determine the existence of stable limit cycles.

We first describe the structure of the dynamical equations with parameters adapted to the electrical or electrochemical interpretation. Thereafter, we calculate two different measurements related to a point in the current–voltage curve. One is impedance spectroscopy by a small ac perturbation of angular frequency ω.^17^ The other is voltage evolution under a large perturbation from the initial equilibrium. We show the phase plane trajectories and bifurcation behavior according to the properties of the impedance spectra.^47−50^ Finally, we discuss the impedance properties that will be necessary in artificial neurons.

We formulate the FHN model equations as follows.^17,57^
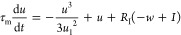
1
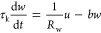
2The physical variables are the membrane voltage *u* and current *I* and an internal recovery current *w*, which represents the effects of changes in ion-channel conductances that occur when the external variables are displaced to a different level. Time is reported in seconds, voltage in volts, current in amperes, and impedance in ohms. In the Supporting Information, we compare different systems of equations and parameters for the FHN model.

The independent parameters in the model of eqs 1 and 2 are voltage response time τ_m_, recovery current response time τ_k_, channel resistor *R*_I_, recovery current resistor *R*_w_, modulation constant *b*, and a reference voltage *u*_1_. As *w* is subjected to a slow relaxation process in eq 2, it is expected that τ_k_ ≫ τ_m_ in the application of the model as an excitable neuron membrane.^58^

A series of derived parameters are useful for the physical interpretation: membrane capacitance, EC resistances, recovery current inductor, ratio of time scales, and ratio of resistances.
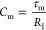
3
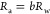
4
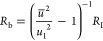
5
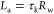
6
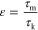
7
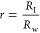
8In a steady state situation (indicated by an overbar), the model provides a stationary current–voltage (*I*–*u*) with the following expression
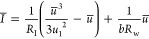
9

Figure 1 presents the *I*–*u* curves and the phase plane of *u* and *w* for two different sets of parameters. The nullclines *ẇ* = 0 and *u̇* = 0 have the expressions
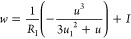
10
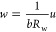
11and the fixed points correspond to their intersections.

**Figure 1:**
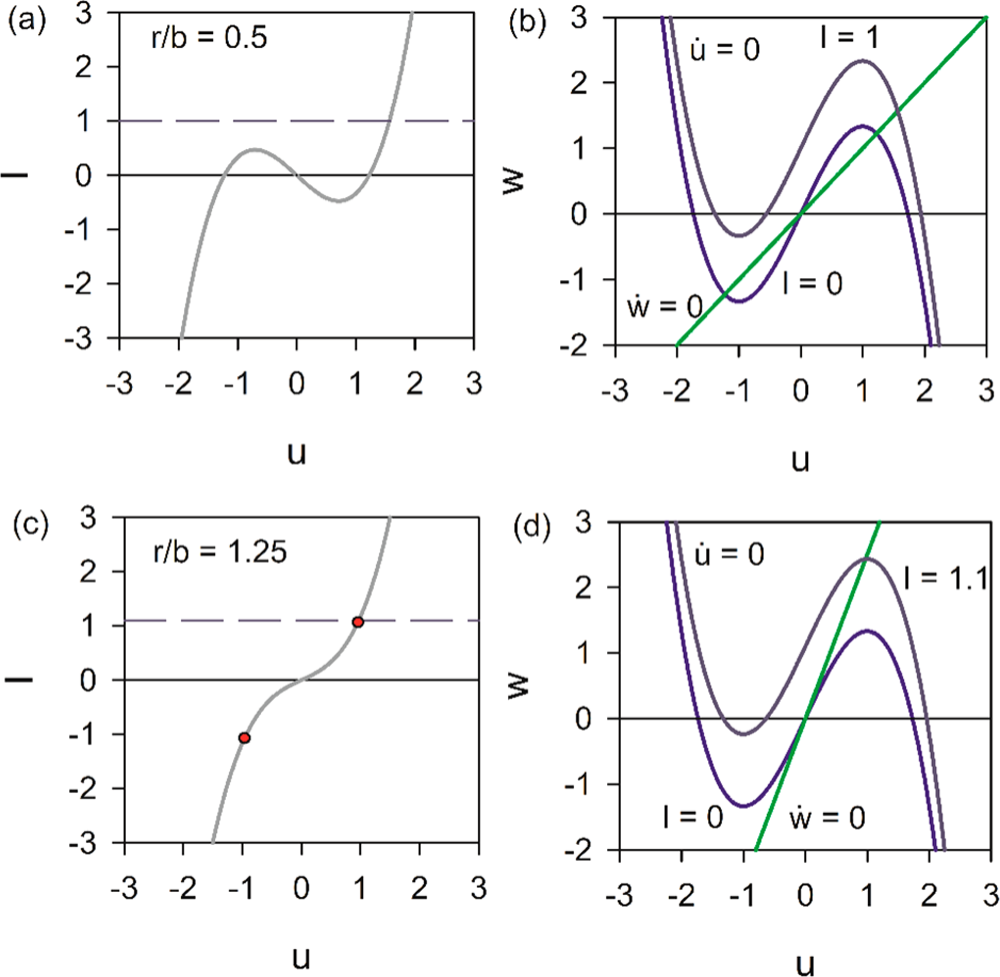
Current–voltage stationary curve and the corresponding phase plane. *R*_I_ = 0.5, *b* = 0.8, ε = 0.1, *u*_1_ = 1, and two values of current as indicated (a and b) *r* = 0.4, and (c and d) *r* = 1. The red points in panel c indicate the Hopf bifurcations: *u*_H_ = ±0.9591, and *I*_H_ = ±1.0678.

To obtain the response to a small perturbation, we develop linearly eqs 1 and 2, with small quantities indicated by the tilde. To calculate the ac impedance *Z* = *ũ*/*Ĩ*, we take the Laplace transform, d/d*t* → *s*, where *s* = *i*ω. Hence
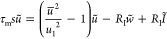
12
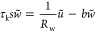
13The result is
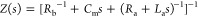
14

The EC corresponding to eq 14 is shown in Figure 2a. It recovers the inductor introduced by Nagumo et al.^23^ However, in contrast to Nagumo’s circuit that contains a tunnel diode element, Figure 2a is for a small perturbation measurement and it is fully described by resistances, capacitors, and inductors that are only a function of the voltage as indicated in eqs 3–6.

**Figure 2:**
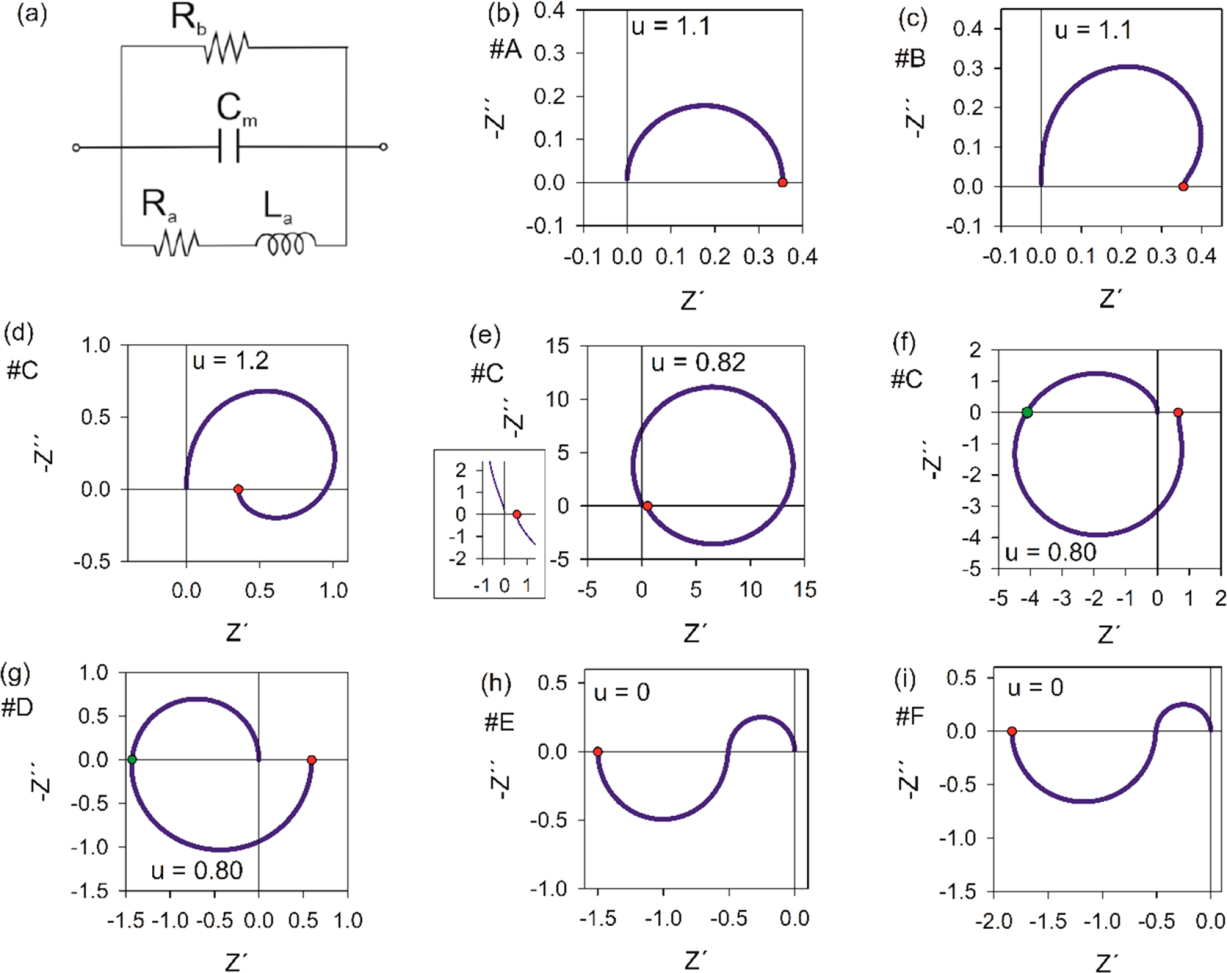
(a) Small ac EC for the FHN model. (b–i) Impedance spectra for different models at the indicated voltages. Panel e shows an amplification of the region close to the origin.

Hereafter we set *u*_1_ = 1 and remove the overbar; hence, eq 9 gives
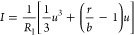
15The dc resistance *R*_dc_ = *Z*(ω = 0) is
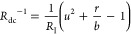
16The EC in Figure 2a is the same as that obtained previously for a memristive element and an integrate-and-fire neuron.^17^ However, in the FHN model, there is always a negative resistance *R*_b_ in the system [in the voltage range −1 < *u* < 1 (eq 5)], corresponding to the membrane intrinsic characteristic, that provides the built-in firing mechanism. The effect of the current of recovery variable *w* is an added positive parallel resistance; hence, the total resistance in eq 16 may be positive or negative. A negative *R*_dc_ occurs when the slope of the blue curve *u̇* = 0 at *u* = 0 in Figure 1 is larger than that of the green curve, which happens when *r*/*b* < 1 (Figure 1a). Here the current is three-valued, although at a higher current it becomes single-valued, indicating a saddle-node bifurcation.

In Figure 2b–i, we plot the impedance spectra generated by the EC in Figure 2a with different sets of parameters listed in Table 1, in the complex plane plot representation, where *Z* = *Z*′ + *iZ*″. The spectra corrrespond to the shapes described previously.^17,59^ All the spectra finish at the origin at ω → ∞ because there is no series resistance.

**Table 1:** Model Parameters with *R*_I_ = 0.5 Ω and τ_m_*=* 10^–2^ s in All Cases

model	*b*	*r*	*r*/*b*	ε	*u*_Hopf_
A	1	1.2	1.2	20	
B	1	1.2	1.2	1.8	
C	1	1.2	1.2	0.316	0.82690
D	1	1.2	1.2	0.01	0.99498
E	1.2	0.8	0.66	0.01	0.99398
F	1.1	0.8	0.73	0.01	0.99448

To analyze the stability of the fixed points, we apply a standard normal mode analysis to the linearized equations in eqs 12 and 13. The Jacobian is
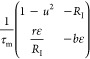
17The eigenvalues λ (in units of τ_m_^–1^) are determined by the following equations
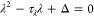
18
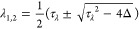
19
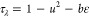
20
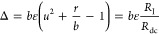
21
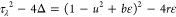
22These quantities are shown in Figure SI1 as a function of voltage. Note that the sign of the determinant coincides with that of the total resistance. Δ < 0 corresponds to real eigenvalues λ_1,2_ with the opposite sign. This is the region of negative *R*_dc_, in which the three fixed points are a saddle and two sinks. The line *r* = *b* is a pitchfork bifurcation. On the contrary, *r*/*b* > 1 corresponds to the single-valued *I*–*u* (Figure 1c) with a positive *R*_dc_. Here the stability of the fixed point is determined by τ_λ_ < 0. τ_λ_ = 0 is the Hopf bifurcation. When τ_λ_ > 0 and τ_λ_^2^ – 4Δ < 0, the fixed point becomes an unstable source with a pair of complex conjugate values λ_1,2_. This behavior produces oscillations and spikes in *u*(*t*).

We examine in the following the different situations according to the bifurcation regimes (these are classified in the Supporting Information), by solving the dynamical equations for the sets of model parameters listed in Table 1, with an initial condition Δ*u* = ±1 with respect to the fixed point. A Mathematica program in the Supporting Information allows one to see the results of calculations for any chosen sets of parameters.

We start with cases of positive dc resistance *r*/*b* > 1. The condition for a loop in the fourth quadrant of the complex plane is τ_k_ > *R*_a_*C*_m_,^17^ which corresponds to
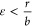
23Figure 2d develops an inductive loop, while panels b and c of Figure 2 do not have such a loop. Model A in Figure 3a has ε ≫ *r*/*b*. Here the impedance spectrum is a simple RC arc, and the trajectory (starting at the orange point) simply falls rapidly to the stable fixed point in an ordinary relaxation process. In model B of Figure 3b, the impedance spectrum bends at the real axis but does not cross it as we have ε > *r*/*b*. These examples do not really belong to FHN because ε > 1 makes *w* the fast variable contrary to the normal physical interpretation. We do not expect to obtain these impedance spectra in the FHN neuron, but they are shown for the sake of completeness.

**Figure 3:**
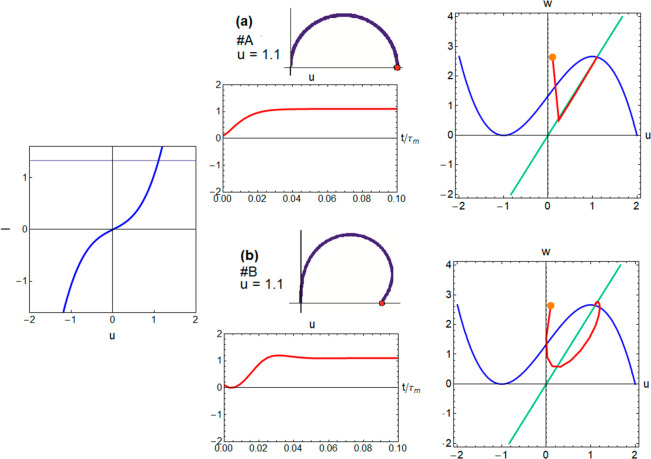
Voltage transients, phase plane trajectories, and impedance spectra for models with different ε values. The *I*–*u* curve is the same in both cases.

In Figure 4, we change to model C with ε < 1 (but not very small). In Figure 4a, the current is well above the Hopf bifurcation; hence, the fixed point is a sink. Now the inductive feature in the fourth quadrant of the complex plane has been fully developed, as ε < *r*/*b* is satisfied. In Figure 4b, the current is closer to the Hopf bifurcation but in the stable side, and the trajectory spirals around the fixed point and falls to it in a damped oscillation *u*(*t*).

**Figure 4:**
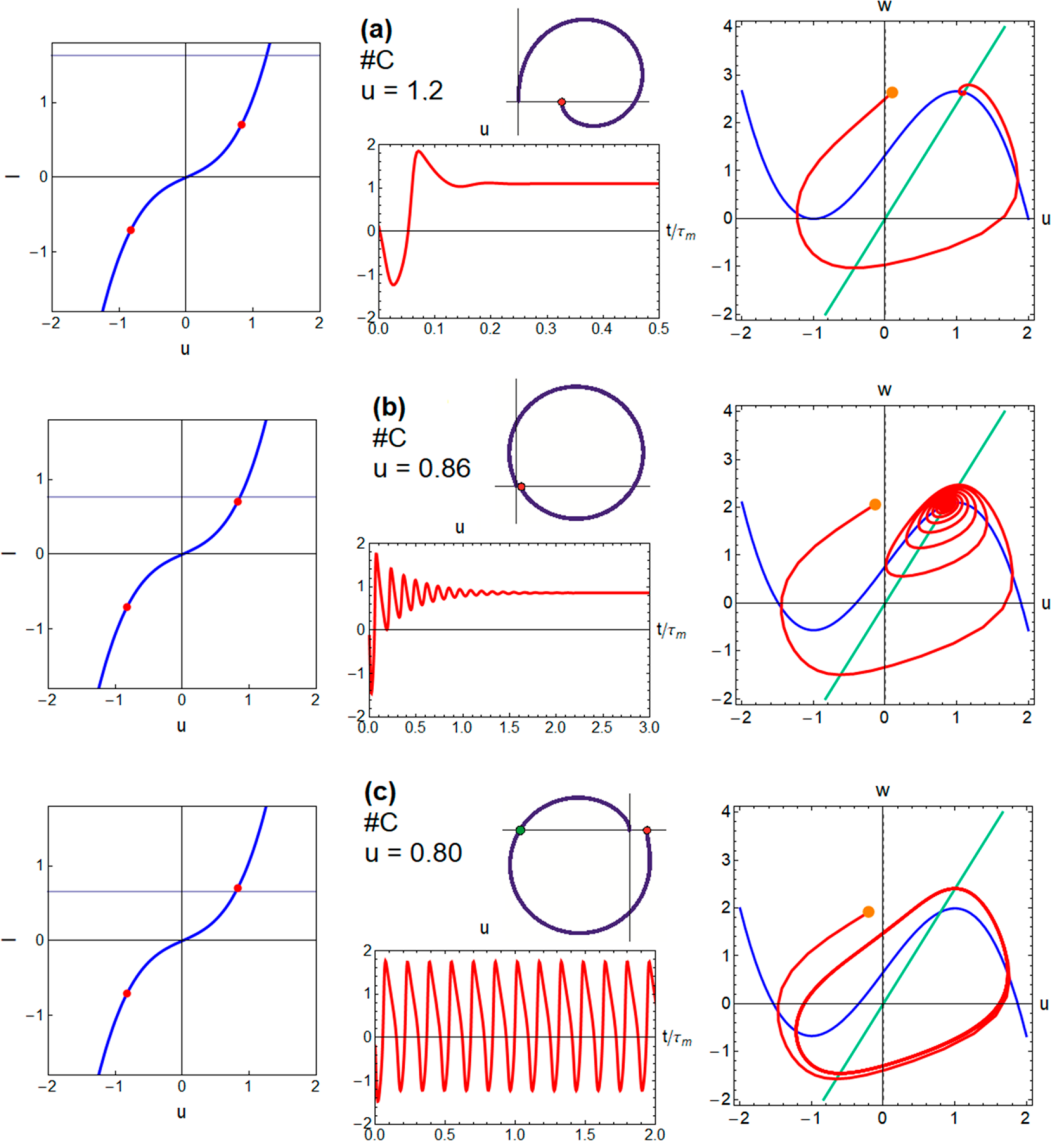
Voltage transients, *I*–*u* curves (red points indicate the Hopf bifurcations), phase plane trajectories, and impedance spectra for model C at different voltages.

In Figure 4c, the Hopf bifurcation has been overcome. The trajectories in the phase plane change suddenly from a decay to the sink to a track around the source fixed point that never decays to it, in a stable limit cycle. The impedance pattern also changes significantly (Figure 2f,g), due to the combination of the two parallel resistive branches, a fast mode with negative resistance and a slow mode with positive resistance, the latter controlled by the series inductor.^47^ Hence, the real part of the impedance becomes negative at high frequencies, in the intercept indicated by a green point, while it is positive at low frequencies and equal to the positive *R*_dc_. This is the hidden negative impedance introduced by Koper.^59^ This pattern corresponds to the limit cycle oscillations that induce neuron spiking.

In model D we change to ε ≪ 1. In contrast to the spirals of Figure 4 obtained for ε ≈ 1, in Figure 5 the trajectories in the phase plane become relaxation oscillations, in which the motions consist of fast horizontal portions where only *u* changes and the pieces of the slow mode in which *w* follows eq 10.

**Figure 5:**
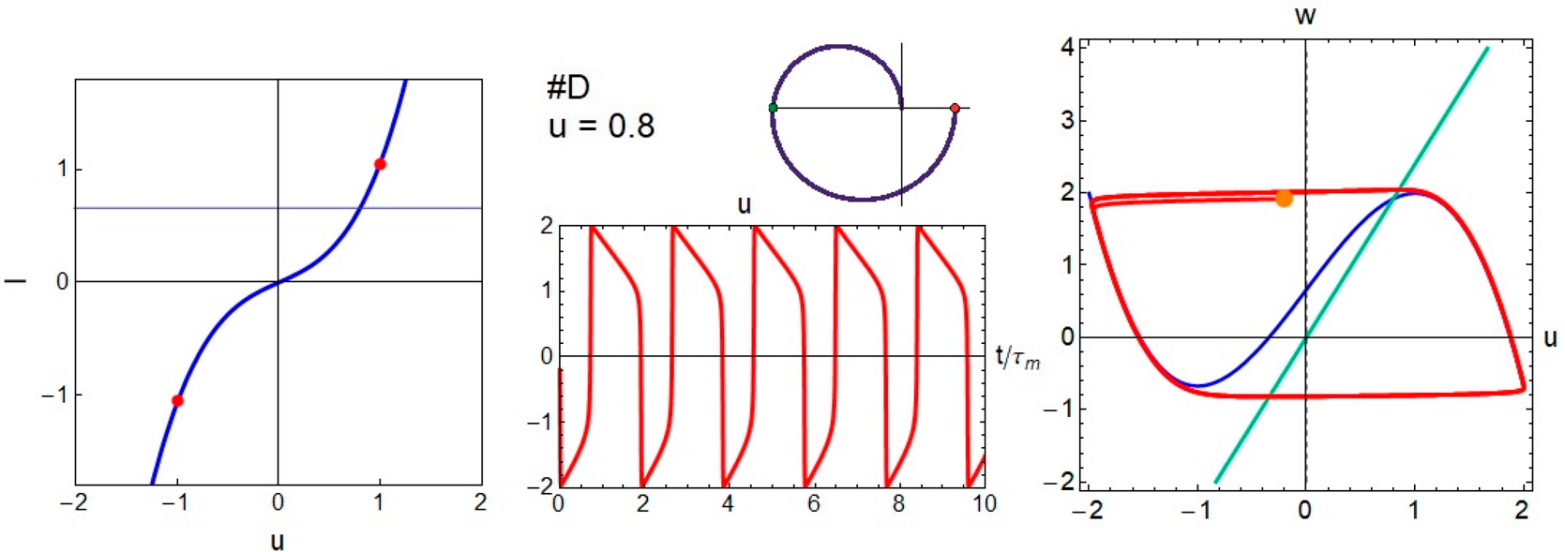
Voltage transient, *I*–*u* curve (red points indicate the Hopf bifurcations), phase plane trajectory, and impedance spectra for model D.

While the trajectories are discussed with respect to time, we can obtain further information about the system by analyzing the dependence of the EC elements with respect to frequency, noting that small frequencies correspond to the dynamics of longer times, and vice versa. This is shown in Figure SI2.

Finally, we take the cases in which *r*/*b* < 1 and Δ < 1, implying that the central fixed point is an unstable saddle (Figure 6). Here *R*_dc_ < 0; hence, the impedance at low frequency starts in the negative axis of the complex plane (Figure 2h,i). The system initially sitting at the unstable *u* = 0 will jump immediately to the either of the other equilibrium points that are sinks, depending on the basin of attraction in which the initial condition sits, as shown for model E in Figure 6a. Furthermore, *I*(*u*) can be measured only as a transient behavior, so that the impedance at *u* = 0 cannot be measured, unless we add a series resistances and turn the system into one in which the negative impedance is hidden as explained above. The intrinsic instability creates a rich set of dynamics. For example, a small modification from model E to model F displaces the Hopf bifurcation so that the fixed points become unstable, and the system enters limit cycle oscillations around *u* = 0 as shown in Figure 6b. Meanwhile, the impedance spectrum in Figure 6b is quite similar to the previous case.

**Figure 6:**
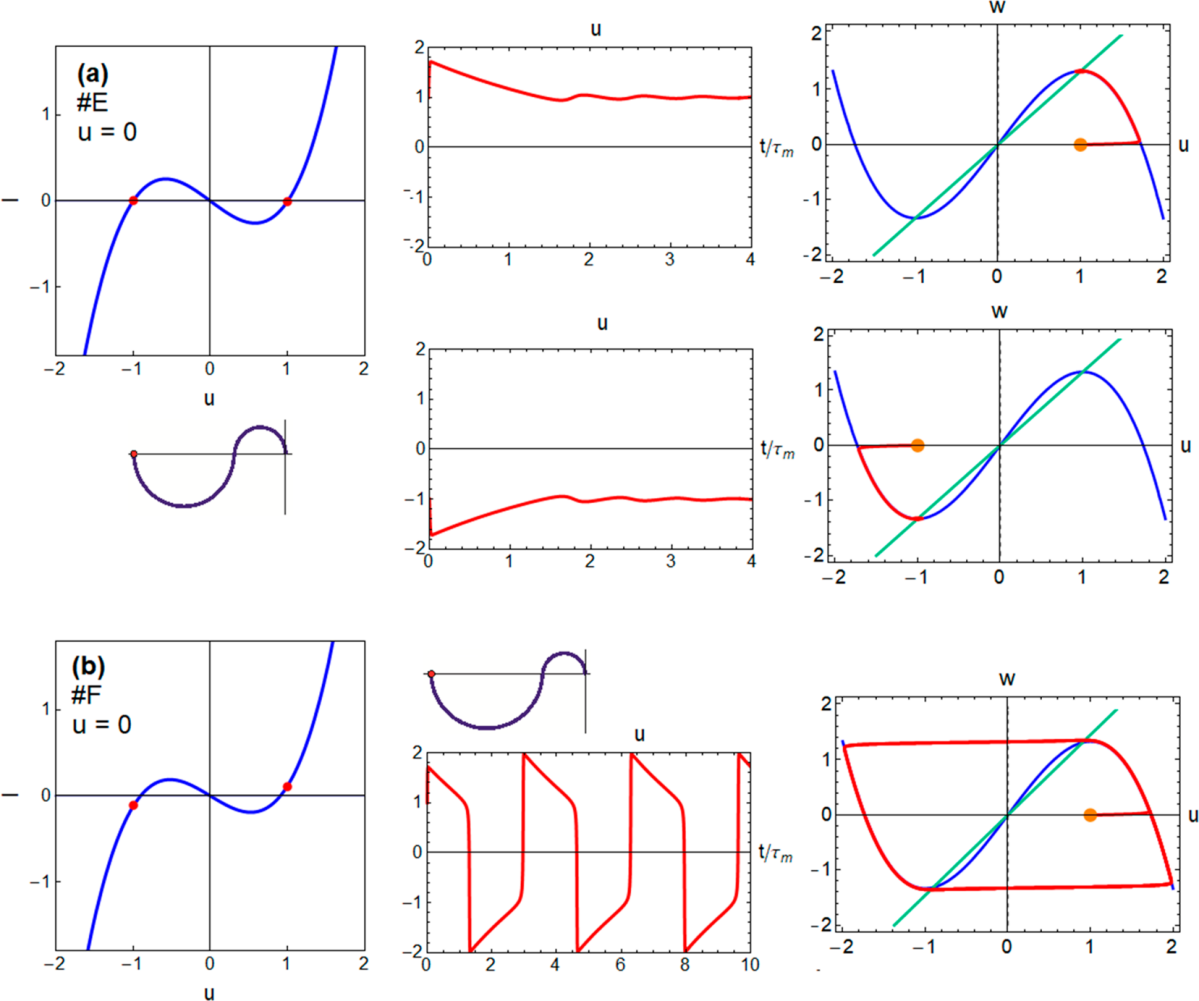
Voltage transients, *I*–*u* curves (red points indicate the Hopf bifurcations), phase plane trajectories, and impedance spectra for (a) model E with two different initial conditions and (b) model F.

We have classified the dynamic regimes of the FHN neuron and the associated EC and impedance spectra. The main result is that a neuron element needs three internal components: capacitor, inductor, and negative differential resistance (NDR).

The membrane capacitance is well-known, due to the polarization by different ionic concentrations at the two sides.

The inductor element requires some explanation, as this type of inductor is unrelated to the storage of energy in a magnetic field. Recently, we showed that the inductor element is generally explained by the relaxation of the slow variable in eq 2.^17^ The identification of an inductive process in the nerve membrane dates back to 1940.^60^ The induction mechanism was explained by HH^18^ when they proposed that the potassium conductance is proportional to a power of a variable that obeys a first-order equation, to match the very different transient curves: the delayed increase in depolarization but simple exponential decay in repolarization. Hodgkin explains very clearly in his biography^61^ that “the inductance is mainly due to the delayed increase in potassium conductance which can make membrane current lag behind voltage provided the internal potential is positive to the potassium equilibrium potential.” This interpretation of the inductor was supported by later measurements.^62^ However, some present discussions^63^ of the inductor still aim to find in neurons a magnetic coil or a piezoelectric effect. These physical properties are not needed because even minimal dynamical models generate the inductor as explained above.

Finally, the NDR is a required condition for the rhythmic oscillations, as it has been clearly recognized in the electrochemical reactions^49^ and in biological neurons.^64,65^

These insights can be used for the construction of artificial neurons. On the basis of our operational understanding at the EC level, we can turn to the construction of a device and ensure that it delivers the specified operation by the reproduction of the frequency domain behavior of the target application. It is encouraging that the two first elements of the EC of a minimal neuron, namely, capacitor and inductor, have been observed in halide perovskite memristors, where the inductive spectra of Figure 2d have been clearly obtained.^54^ However, our results indicate the necessity of building a NDR element in the device.

In conclusion, we developed the small ac impedance that allows us to calculate the characteristic spectra of different dynamical regimes of the FitzHugh–Nagumo neuron. We could thus obtain the basic impedance response and the equivalent circuit of a minimal dynamical neuron model. Our results show the basic electrical structure required, consisting of some fundamental elements that give the neuron dynamics: capacitor, inductor, and at least one negative resistance. The impedance spectra immediately reveal the stability, excitability, and bifurcation properties according to the regimes of model parameters and the nature of stationary points. Here we have obtained the impedance results from a previous complete understanding of the parameters and dynamical equations. In practical operation, one can measure the impedance and reach conclusions about the physical properties based purely on the shape of experimental impedance spectra, avoiding the need for highly specific modeling. The measurement of impedance spectroscopy has the potential to become an important tool in the study of real neurons and for candidates for ionic/electronic devices to artificial spiking neurons, complementary to the usual study of transient and periodic phenomena in the time domain.

## Supporting Information Available

Expression of the mathematical model, bifurcation parameters, interpretation of impedance elements, Mathematica program for simulation, and graphical representation (PDF)
