# Supplementary material for: A Frequency Domain Analysis of the Excitability and Bifurcations of the FitzHugh–Nagumo Neuron Model
Source: J Phys Chem Lett. 2021 Nov 5;12(45):11005–13. doi: 10.1021/acs.jpclett.1c03406 (PMC8709542; doi:10.1021/acs.jpclett.1c03406)
Supplement: Supplementary file 1 — jz1c03406_si_001.pdf [file jz1c03406_si_001.pdf]

Supporting Information

A Frequency Domain Analysis of Excitability and Bifurcations of the Fitzhugh-Nagumo Neuron Model

Juan Bisquert*
Institute of Advanced Materials (INAM), Universitat Jaume I, 12006 Castelló, Spain
Email: bisquert@uji.es

1. Expression of FitzHugh-Nagumo (FHN) dynamical systems

There are different equivalent expressions of the FHN model. We present the main two representative parametrizations and our own formulation

Izhikewich[1] and Kostova et al.[2]	a, I, b_I, c_I	$\frac{du}{dt} = u(a-u)(u-1) + (-w+I)$ (1)<br>$\frac{dw}{dt} = b_I u - c_I w$ (1)<br>$u(a-u)(u-1) = -u^3 + (1+a)u^2 - au$
Rocsoreanu et al.[3]	c_R, a_R, b_R	$\frac{1}{c_R}\frac{du}{dt} = -\frac{u^3}{3} + u + w$ (2)<br>$c_R \frac{dw}{dt} = -u + a_R - b_R w$ (2)
This work	τ_m, τ_k, u_1 R_I, R_w, I	$\tau_m \frac{du}{dt} = -\frac{u^3}{3u_1^2} + u + R_I(-w+I)$ (3a)<br>$\tau_k \frac{dw}{dt} = \frac{1}{R_w} u - bw$ (3b)

The expressions (1) and (2) are related by a linear transformation of the variables given in p. 12 of the book [3].

Our expression (3) of the dynamical system has been selected so that the physical dimension of each variable is explicit. In this way the electrical quantities of the equivalent circuit are transparent and furthermore the results can be compared with experiments. Therefore, we have in (3)

t is time

u is a voltage

w is electrical current.

It follows that the constants have the dimensions
τ_m, τ_k, are times
u_1 is voltage
I is electrical current.
R_I, R_w are electrical resistances.

The use of τ_m and R_I in the equation for the voltage, 3a, as well as the associated capacitance, $C_m = \tau_m/R_I$, is quite extended,[4-6] since the original interpretation of the Hodgkin-Huxley model in terms of electrical circuits.[7] Therefore our equation for u is quite conventional. In the equation 3b for w we have used a similar structure for the definition of parameters τ_k, R_w that generate the inductance $L_a = \tau_k R_w$.

The number of parameters in our model can be reduced to those that control de dynamical properties by defining dimensionless variables, as we have done in Eq. (7) and (8) of the main text:

$$\varepsilon = \frac{\tau_m}{\tau_k} \qquad (7)$$

$$r = \frac{R_I}{R_w} \qquad (8)$$

However, we prefer to use all the initial parameters so that the meaning of the circuit elements is very easily understood.

In the main text we have set $u_1 = 1$ to simplify the calculations. This restriction is effect is a rescaling of the voltage scale. A general u_1 needs to be restored when comparing with experiments.

2. Bifurcation parameters

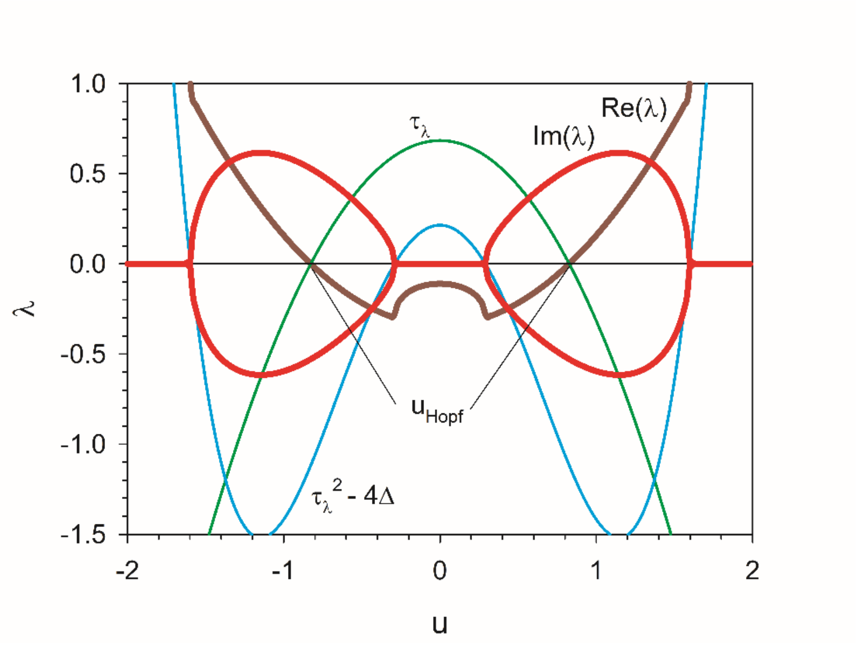

Fig. SI1. Bifurcation parameters for model #C.

Model	b	r	r/b	ε	u_{Hopf}
#C	1	1.2	1.2	0.316	0.82690

$\Delta < 0$ corresponds to real eigenvalues $\lambda_{1,2}$ with opposite sign. This is the region of negative R_{dc}, the three fixed points are a saddle and two sinks. The line $r = b$ is a pitchfork bifurcation. On the other hand $r/b > 1$ corresponds to the single valued $I - u$, Fig. 1c. Here the stability of the fixed point is determined by $\tau_\lambda < 0$. At $\tau_\lambda = 0$ is the Hopf bifurcation. When $\tau_\lambda > 0$ and $\tau_\lambda^2 - 4\Delta < 0$ the fixed point becomes an unstable source with a pair of complex conjugate $\lambda_{1,2}$.

Summary:

$\frac{r}{b} > 1$	R_{dc} positive	
$1 - u^2 - b\varepsilon = 0$	Hopf bifurcation	
$\varepsilon < \frac{r}{b}$ (also) $\tau_k > R_a C_m$	Loop in fourth quadrant	

$\frac{r}{b} < 1$	R_{dc} negative	

3. Interpretation of impedance elements

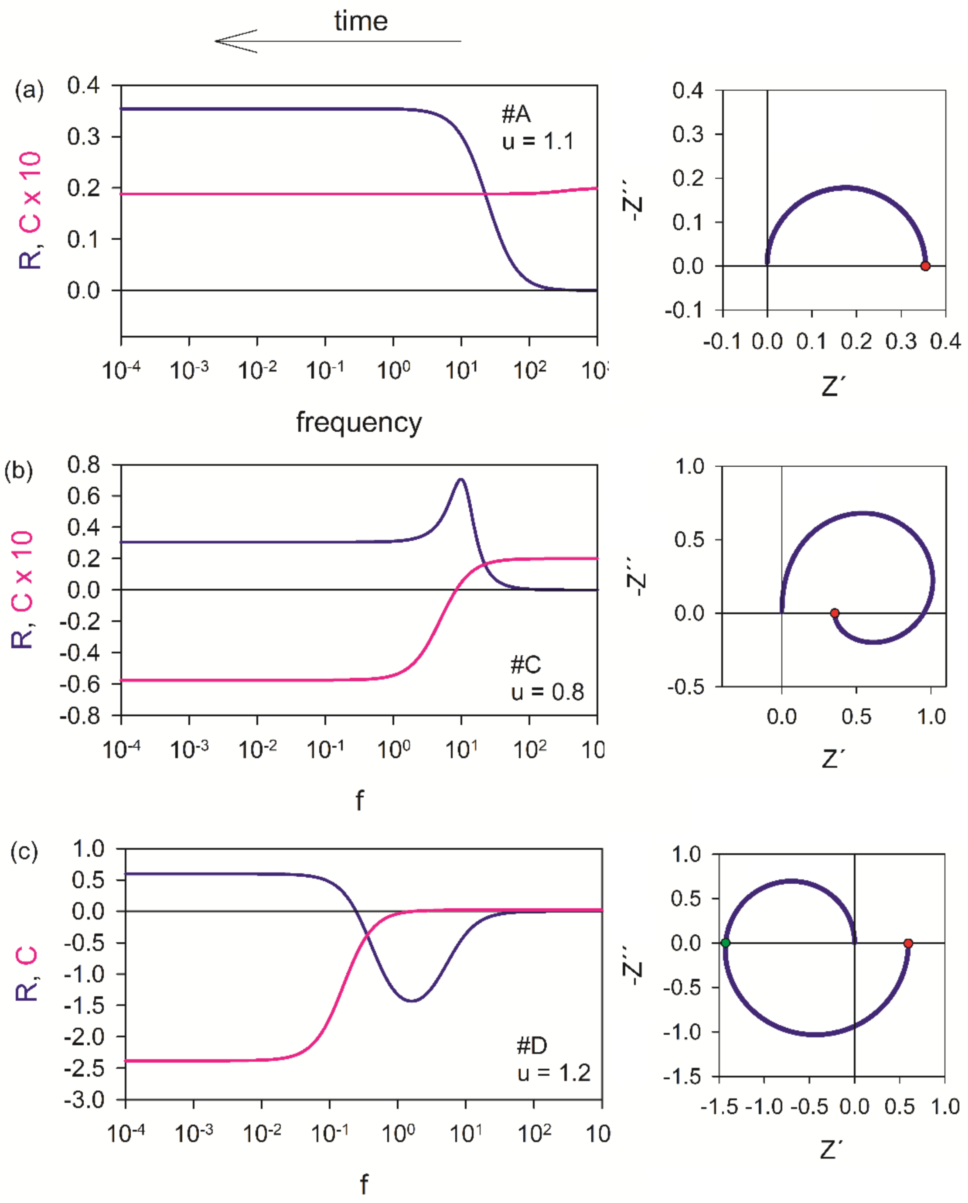

Fig. S2. Representation of the resistance $R(\omega) = \mathrm{Re}[Z(\omega)]$ and capacitance $C(\omega) = \mathrm{Re}[1/i\omega Z(\omega)]$ as a function of the frequency $f = \omega/2\pi$. $R_l = 0.5$ and $C_m = 0.02$.

We analyze the dependence of the EC elements with respect to frequency, noting that small frequencies correspond to the dynamics of longer times, and viceversa. In Fig. S2 we calculate a total resistance and capacitance. For the simple arc spectrum in Fig. S2a the resistance increases suddenly at the characteristic frequency/time of the relaxation, given by RC or its inverse. In the spectrum with an inductive arc and positive resistance,[8] Fig. S2b, the low frequency resistance is smaller than the intermediate frequency resistance which has been used to explain hysteresis effects in solar cells.[9] In the oscillatory circuit S2c the variation of resistance is inverted with respect to b: a minimum

of negative resistance that turns to positive at long times or low frequencies. Note that both (b) and (c) display a region of effective negative capacitance at low frequency.[8,10]

References

(1) Izhikevich, E. M. *Dynamical Systems in Neuroscience*; MIT Press, 2007.

(2) Kostova, T.; Ravindran, R.; Schonbek, M. Fitzhugh–Nagumo revisited: types of bifurcations, periodical forcing and stability regions by a Lyapunov functional, *International Journal of Bifurcation and Chaos* **2004**, *14*, 913-925.

(3) Rocşoreanu, C.; Georgescu, A.; Giurgiţeanu, N. *The FitzHugh-Nagumo model: bifurcation and dynamics*; Kluwer Academic Publishers, 2000.

(4) Brette, R.; Gerstner, W. Adaptive exponential integrate-and-fire model as an effective description of neuronal activity., *J Neurophysiol.* **2005**, *94*, 3637-3642.

(5) Jolivet, R.; Lewis, T. J.; Gerstner, W. Generalized integrate-and-fire models of neuronal activity approximate spike trains of a detailed model to a high degree of accuracy, *Journal of Neurophysiology* **2004**, *92*, 959-976.

(6) Gerstner, W.; Kistler, W. M.; Naud, R.; Paninski, L. *Neuronal Dynamics. From Single Neurons to Networks and Models of Cognition*; Cambridge University Press, 2014.

(7) Hodgkin, A. L.; Huxley, A. F. A quantitative description of membrane current and its application to conduction and excitation in nerve, *J Physiol* **1952**, *117*, 500-544.

(8) Mora-Seró, I.; Bisquert, J.; Fabregat-Santiago, F.; Garcia-Belmonte, G.; Zoppi, G.; Durose, K.; Proskuryakov, Y. Y.; Oja, I.; Belaidi, A.; Dittrich, T.; Tena-Zaera, R.; Katty, A.; Lévy-Clement, C.; Barrioz, V.; Irvine, S. J. C. Implications of the negative capacitance observed at forward bias in nanocomposite and polycrystalline solar cells, *Nano Lett.* **2006**, *6*, 640-650.

(9) Bisquert, J.; Guerrero, A.; Gonzales, C. Theory of hysteresis in halide perovskites by integration of the equivalent circuit, *ACS Phys. Chem Au* **2021**, *1*, https://doi.org/10.1021/acsphyschemau.1021c00009.

(10) Klotz, D. Negative capacitance or inductive loop? – A general assessment of a common low frequency impedance feature, *Electrochem. Comm.* **2019**, *98*, 58-62.

Mathematica calculations

```
(* Model Parameters*)
uapp = 0.1; RI = 0.5; b = 1; r = 1.2; ϵ = 0.316; r / b
Tm = 10^(-2); τm = Tm; τk = τm / ϵ ;
ΔV = 1; vα = 0;  t1 = 2; V1 = -2; w0 = (uapp - vα) / (b Rw); v0 = uapp + ΔV;
wrep0 = -2; wrep1 = 4;
Rw = RI / r;
Ic[up_] := (up^3 / 3 - up) / RI + (up - vα) /  (b Rw);
Iapp = Ic[uapp]
(* bifurcation parameters*)
SR[u_] := ((1 - u^2) + b ϵ)^2 - 4 ϵ r;
Solve[SR[uSR] == 0, uSR]
tau[u_] := 1 - u^2 - b ϵ
Solve[tau[uHo] == 0, uHo];
(* Hopf point *)
uH = N[Solve[tau[uHo] == 0, uHo][[2, 1, 2]]]
IcHo = Ic[uH]

(* Plot of the IV curve*)
tm1 = ParametricPlot[{uvi, Ic[uvi]}, {uvi, -3, 3}, PlotRange → {{-3, 3}, {-2, 3}},
   FrameLabel → {Style["u", FontSize → 20], Style["I", FontSize → 20], "", ""},
   Frame → True, FrameTicks → {{-2, -1, 0, 1, 2}, {-10, -1, 0, 1, 10}, None, None},
   PlotStyle → {{AbsoluteThickness[3], Blue}}, LabelStyle → (FontFamily → "Arial"),
   AspectRatio → 1, ImageSize → 90 × 4`];
tm2 = Plot[{Iapp}, {x, -3, 3}];
tm3 = Graphics[{PointSize[0.03], Red, Point[{-uH, Ic[-uH]}]}];
tm4 = Graphics[{PointSize[0.03], Red, Point[{uH, Ic[uH]}]}];
Show[tm1, tm2, tm3, tm4, PlotRange → {{-2, 2}, {-1.8, 1.8}},
 BaseStyle -> {FontSize → 16}]

(* Plot of u(t)  *)
sol = NDSolve[{
    τm v'[t] == v[t] - v[t]^3 / 3 + RI (-w[t] + Iapp),
    τk w'[t] == (v[t] - vα) / Rw - b w[t],
    v[0] == v0, w[0] == w0}, {v, w}, {t, 0, 10}];
Show[Plot[{Evaluate[{v[t]} /. sol]}, {t, 0, 10}, PlotRange → {{0, t1}, {V1, 2}},
  PlotStyle → {{AbsoluteThickness[3], CMYKColor[0, 1, 1]}},
  BaseStyle -> {FontSize → 16}, Frame → True, LabelStyle → (FontFamily → "Arial"),
  AxesLabel → {Style["t/τm", FontSize → 18], Style["u", FontSize → 18]}]]

(* Plot of the nullcline curves for v and w *)
Show[ContourPlot[{v - v^3 / 3 + RI (-w + Iapp) == 0, (v - vα) / Rw - b w == 0, v == 0},
  {v, -2, 2}, {w, wrep0, wrep1}, Axes → True, BaseStyle -> {FontSize → 18},
  LabelStyle → (FontFamily → "Arial"),
  AxesLabel → {Style["u", FontSize → 20, Black], Style["w", FontSize → 20, Black]},
  ImageSize → 90 × 4`,
  ContourStyle → {{Thickness[0.008], CMYKColor[1, 1, 0]},
    {Thickness[0.008], CMYKColor[1, 0.25, 0.5]}, Dashed}], sol = NDSolve[{
```

```
   τm v'[t] == v[t] - (v[t]^3) / 3 + RI (-w[t] + Iapp),
   τk w'[t] == (v[t] - vα) / Rw - b w[t],
   v[0] == v0, w[0] == w0}, {v, w}, {t, 0, 10}];
 Show[ParametricPlot[{Evaluate[{v[t], w[t]} /. sol]}, {t, 0, 10},
   PlotStyle → {{AbsoluteThickness[3], CMYKColor[0, 1, 1]}},
   BaseStyle -> {FontSize → 18}, PlotRange → {{-0.5, 10}, {-3, 3}, {-3, 3}}]],
 oo2 = Graphics[{PointSize[0.04], Orange, Point[{v0, w0}]}]]

(* Impedance *)
up = uapp; po = 0.5;
Rb[up_] := RI / (up^2 - 1);
Ra = b Rw; La = Rw τk; Cm = τm / RI;
Rdc[up_] := 1 / (1 / Ra + 1 / Rb[up]);
ZI[up_, w_] := 1 / (1 / Rb[up] + Cm I w + 1 / (Ra + La I w))
Ic[up]
Rb[up]
Rdc[up]
ty1 = ParametricPlot[{Re[ZI[up, 2 π 10^pote]], -Im[ZI[up, 2 π 10^pote]]},
   {pote, -4, 4}, PlotRange → {{-1, 1}, {-1, 1}},
   FrameLabel → {Style["Re Z", FontWeight → "Bold"],
     Style["-Im Z", FontWeight → "Bold"], "", ""}, Frame → True,
   FrameTicks → {{-1, 0, 1}, {-1, 0, 1}, None, None},
   PlotStyle → {{AbsoluteThickness[2]}}, AspectRatio → 1, ImageSize → 100 × 4`];
ty2 = Graphics[{PointSize[0.02], Cyan, Point[{Re[ZI[up, 0]], -Im[ZI[up, 0]]}]}];
Show[ty1, ty2, PlotRange → {{-10^po, 10^po}, {-10^po, 10^po}}]

(* Bifurcation parameters*)
λ1[u_] := 1 / 2 (-tau[u] + SR[u]^0.5)
λ2[u_] := 1 / 2 (-tau[u] - SR[u]^0.5)

op1 = ParametricPlot[{uvi, Im[λ1[uvi]]}, {uvi, -3, 3}, PlotRange → {{-3, 3}, {-1, 1}},
   FrameLabel → {Style["u", FontWeight → "Bold"], Style["λ", FontWeight → "Bold"],
     "", ""}, Frame → True, FrameTicks → {{0, 0.5, 1, 1.5, 2}, {0, 1, 10}, None, None},
   PlotStyle → {{AbsoluteThickness[2], Red}}, AspectRatio → 0.5, ImageSize → 70 × 4`];
op2 = ParametricPlot[{uvi, Im[λ2[uvi]]}, {uvi, -3, 3}, PlotRange → {{-3, 3}, {-1, 1}},
   FrameLabel → {Style["u", FontWeight → "Bold"], Style["λ", FontWeight → "Bold"],
     "", ""}, Frame → True, FrameTicks → {{0, 0.5, 1, 1.5, 2}, {0, 1, 10}, None, None},
   PlotStyle → {{AbsoluteThickness[2], Blue}}, AspectRatio → 0.5,
   ImageSize → 100 × 4`];
op3 = ParametricPlot[{uvi, Re[λ1[uvi]]}, {uvi, -3, 3}, PlotRange → {{-3, 3}, {-1, 1}},
   FrameLabel → {Style["u", FontWeight → "Bold"], Style["λ", FontWeight → "Bold"],
     "", ""}, Frame → True, FrameTicks → {{0, 0.5, 1, 1.5, 2}, {0, 1, 10}, None, None},
   PlotStyle → {{AbsoluteThickness[2], Blue}}, AspectRatio → 0.5,
   ImageSize → 100 × 4`];
op4 = ParametricPlot[{uvi, tau[uvi]}, {uvi, -3, 3}, PlotRange → {{-3, 3}, {-1, 1}},
   FrameLabel → {Style["u", FontWeight → "Bold"], Style["λ", FontWeight → "Bold"],
     "", ""}, Frame → True, FrameTicks → {{0, 0.5, 1, 1.5, 2}, {0, 1, 10}, None, None},
   PlotStyle → {{AbsoluteThickness[2], Green}}, AspectRatio → 0.5,
   ImageSize → 100 × 4`];
op5 = ParametricPlot[{uvi, SR[uvi]}, {uvi, -2, 2}, PlotRange → {{0, 2}, {-1, 1}},
   FrameLabel → {Style["u", FontWeight → "Bold"], Style["I", FontWeight → "Bold"],
     "", ""}, Frame → True,
   FrameTicks → {{0, 0.5, 1, 1.5, 2}, {0, 1, 10}, None, None},
   PlotStyle → {{AbsoluteThickness[2], Yellow}}, AspectRatio → 0.5,
   ImageSize → 100 × 4`];
Show[op1, op2, op3, op4, op5, PlotRange → {{-2, 2}, {-1, 1}}]
```

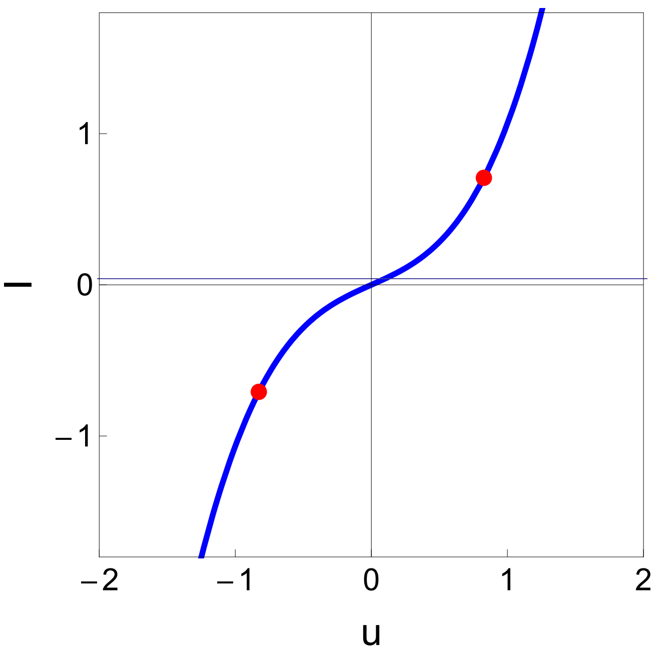

NDSolve::mxst : Maximum number of 10000 steps reached at the point t == 7.783022627314567`. ≫

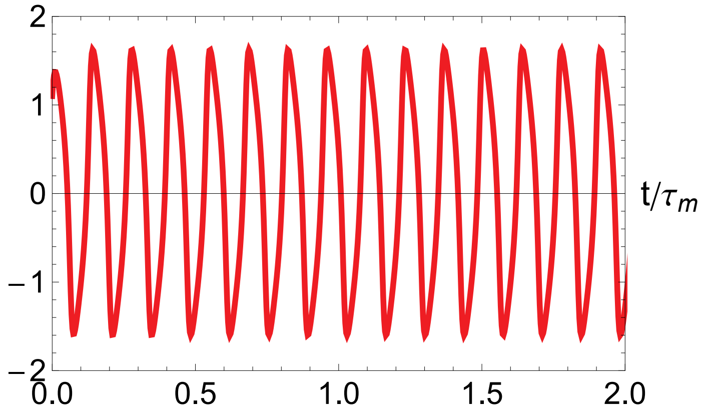

NDSolve::mxst : Maximum number of 10000 steps reached at the point t == 7.783022627314567`. ≫

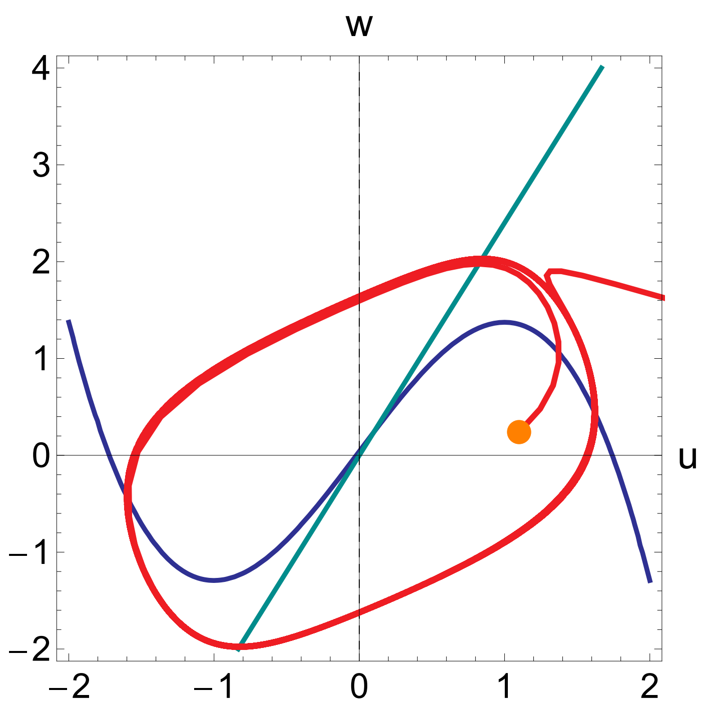


```
0.0406667

-0.505051

2.38095
```

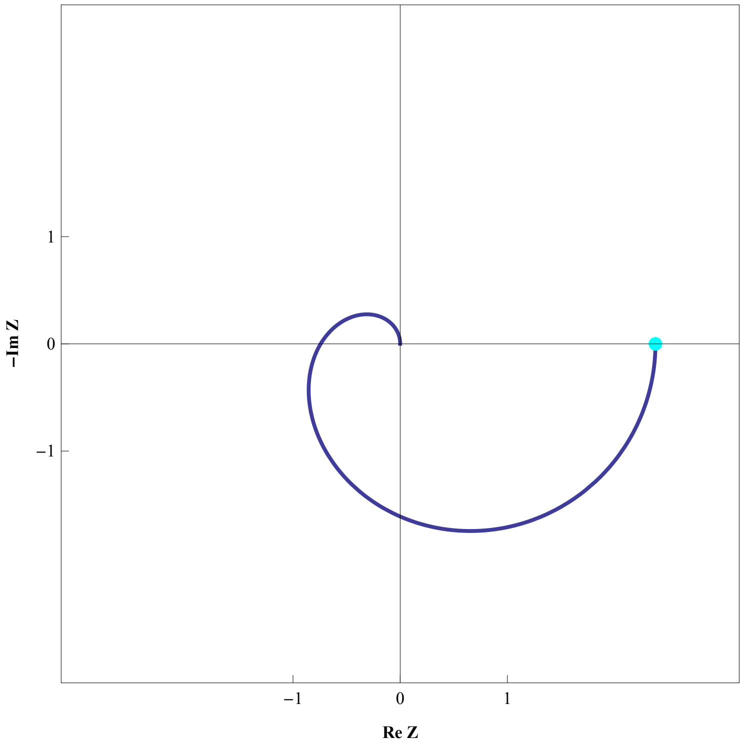

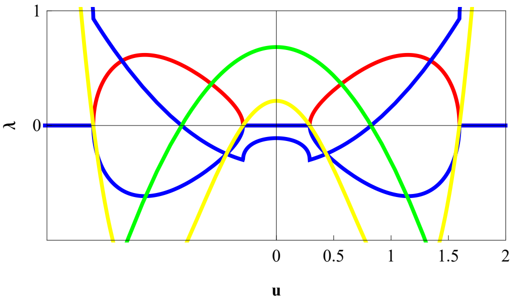
1
0
λ
0
0.5
1
1.5
2
u

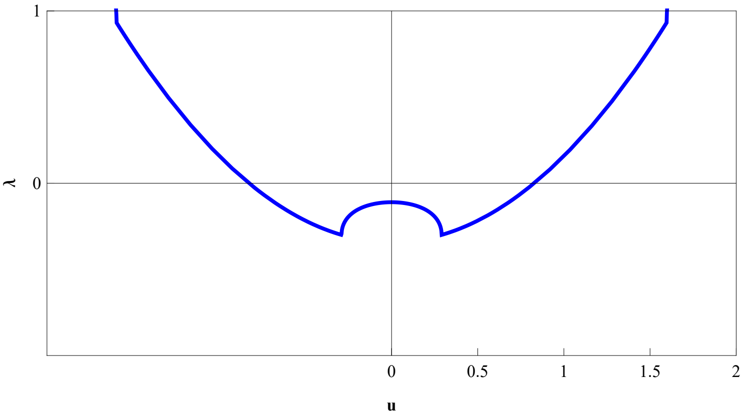
1
0
λ
0
0.5
1
1.5
2
u
